# Resolution of cast nephropathy following free light chain removal by haemodialysis in a patient with multiple myeloma: a case report

**DOI:** 10.1186/1752-1947-2-380

**Published:** 2008-12-09

**Authors:** Kolitha Basnayake, Colin Hutchison, Dia Kamel, Michael Sheaff, Neil Ashman, Mark Cook, Heather Oakervee, Arthur Bradwell, Paul Cockwell

**Affiliations:** 1 Queen Elizabeth Medical Centre, University Hospital Birmingham, Edgbaston, Birmingham, B15 2TH, UK; 2 Barts and the London NHS Trust, Whitechapel, London, E1 1BB, UK; 3 Division of Immunity and Infection, University of Birmingham, Edgbaston, Birmingham, B15 2TT, UK

## Abstract

**Introduction:**

Acute renal failure in multiple myeloma is most frequently caused by cast nephropathy, when excess monoclonal free light chains co-precipitate with Tamm-Horsfall protein in the distal nephron, causing tubular obstruction. The natural history of cast nephropathy after diagnosis is unknown. This report provides supporting histological evidence that, as serum free light chain concentrations fall, intratubular casts may resolve within weeks.

**Case presentation:**

We report the case of a 61-year-old Caucasian woman who presented with multiple myeloma and dialysis-dependent acute renal failure, with serum kappa free light chain concentrations of 15,700 mg/litre (normal range 3.3 to 19.4 mg/litre). Renal biopsy demonstrated cast nephropathy with waxy casts in distal tubules and collecting ducts. There was an interstitial inflammatory cell infiltrate with diffuse fibrosis and tubular atrophy. Following rehydration, chemotherapy and free light chain removal using high cut-off haemodialysis, free light chain concentrations fell to less than 5% of the starting level (500 mg/litre). A repeat renal biopsy 6 weeks after the first showed resolution of cast nephropathy.

**Conclusion:**

These observations indicate that cast nephropathy can quickly resolve on rapid reduction of monoclonal serum free light chains. This has important implications for the development of treatment strategies aimed at improving renal recovery rates for patients in this setting.

## Introduction

Renal failure in multiple myeloma (MM) is associated with high morbidity and mortality. Approximately 10% of newly diagnosed patients require dialysis. Of these, 80% will not recover renal function [1,2]. The predominant cause of dialysis-dependent renal failure in this setting is cast nephropathy. Monoclonal free light chains (FLCs) are freely filtered by the glomerulus, following which they are reabsorbed and metabolised by the proximal tubule epithelium. When the burden of filtered FLC exceeds this resorptive capacity, FLC will then pass through into the distal nephron. Here co-precipitation with Tamm-Horsfall protein (THP) occurs resulting in intratubular obstruction [3-5].

The natural history of the pathology of cast nephropathy is unknown. There has been one previous report of a follow-up renal biopsy after the initial diagnostic biopsy showing myeloma kidney [6]. This patient was treated with chemotherapy and initially received haemodialysis, converting to continuous ambulatory peritoneal dialysis. The patient became dialysis-independent after 3 months with an associated reduction in serum paraprotein concentration and urinary light chain excretion. A repeat renal biopsy at 8 months showed no cast nephropathy.

Only one recent study has accurately assessed the kinetics of FLCs in patients with severe renal failure [7]. This report indicated that serum FLC concentrations remained elevated for many weeks despite effective induction chemotherapy. It also showed that high cut-off haemodialysis led to rapid reduction in serum FLCs and with effective chemotherapy, this reduction is maintained.

We report a case of a patient with cast nephropathy which resolved within 6 weeks after treatment with chemotherapy and high cut-off haemodialysis.

## Case presentation

A 61-year-old Caucasian woman presented to her general practitioner complaining of feeling tired and weak. She had previously been fit and well, and did not take any medications. Initial investigations revealed that she was in acute renal failure with a serum creatinine of 872 μmol/litre and serum urea of 31.5 mmol/litre. Serum calcium and urate levels were normal. Haemoglobin concentration was 7.8 g/dl (78 g/litre). Urine output was approximately 2 liters/day.

**Figure 1:**
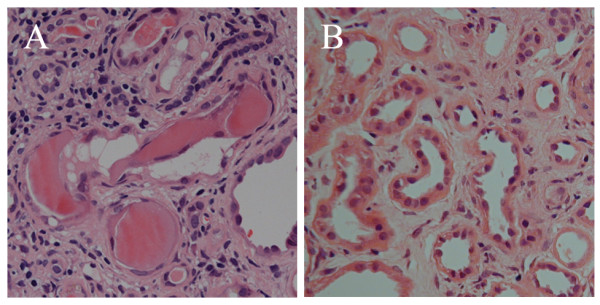
**Renal biopsies**. (A) High power haematoxylin and eosin stained section of the first biopsy showing hard, fractured casts with associated giant cell reaction. There is a peritubular inflammatory cell infiltrate, with significant interstitial fibrosis and tubular atrophy. (B) High power haematoxylin and eosin stained section of the second biopsy demonstrating resolution of myeloma casts. There is partial resolution of the interstitial inflammatory infiltrate. The degree of interstitial fibrosis and tubular atrophy remained unchanged.

Serum immunofixation electrophoresis identified monoclonal free kappa light chains. FLC concentrations were quantified using a serum immunoassay [8] (FREELITE, The Binding Site, Birmingham, UK): serum kappa 15,700 mg/litre (normal range 3.3 to 19.4 mg/litre) [9], urine kappa 2450 mg/litre, serum lambda 22.4 mg/litre (5.7 to 26.3 mg/litre) [9], kappa/lambda ratio 701 (normal range: 0.26 to 1.65) [9]. Immunoglobulin concentrations were: IgG 6.81 g/litre (6 to 16 g/litre), IgA 0.79 g/litre (0.8 to 4.0 g/litre) and IgM 0.38 g/litre (0.5 to 2.0 g/litre). Lytic lesions were seen on skeletal survey. Bone marrow examination showed 90% plasma cell infiltration. Renal ultrasound was unremarkable. Renal biopsy demonstrated waxy casts consistently affecting approximately 30% of distal tubules and collecting ducts, with associated peritubular inflammatory cell infiltrate (Figure 1A). There was moderate diffuse interstitial fibrosis and tubular atrophy. A diagnosis of multiple myeloma and acute renal failure due to cast nephropathy was made.

For the first 4 weeks, the patient was managed with intravenous hydration. During this time, a 40% reduction in serum FLC concentration to 8590 mg/litre was seen, but she remained in severe renal failure with an eGFR of <10 ml/min/1.73 m^2^. Chemotherapy was then initiated with pulsed high-dose dexamethasone. Each pulse consisted of 40 mg once daily for 4 days.

Thalidomide 100 mg daily was added 7 days later. FLC concentrations were reduced further to 1990 mg/litre 5 days after initiation of the first pulse. FLC removal haemodialysis was commenced at this point, using a high cut-off dialyser with increased permeability to substances up to a molecular weight of 60 kDa [7] (HCO 1100; Gambro Dialysatoren GmbH, Hechingen, Germany).

**Figure 2:**
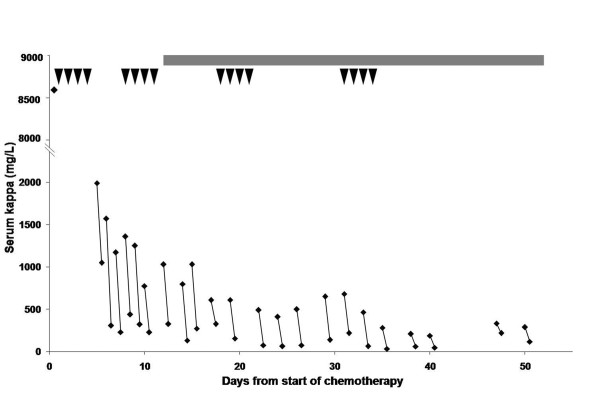
**Serum free kappa light chain concentrations**. Changes in concentration with dialysis and chemotherapy with time are shown. Pre-dialysis and post-dialysis values are connected by lines. The concentration at the start of the first pulse of dexamethasone is also shown. The arrowheads represent daily doses of dexamethasone. Daily thalidomide is indicated by the solid line at the top of the chart.

Two dialysers were used in series as previously described [7]. The patient was dialysed for 3 hours on day 1 and 6 to 8 hours on days 2 to 6. Three further dialysis sessions of 8 hours were performed from days 8 to 14. Following this, the patient continued on a thrice weekly 4 to 6 hour dialysis schedule. Figure 2 presents serum-free kappa concentrations pre- and post-dialysis as well as a summary of the chemotherapy regimen. The median decrease in serum FLC concentration was 74% (range: 35 to 88%) per dialysis session. Albumin, magnesium and calcium losses were replaced when indicated.

After 16 dialysis treatments, 33 days after the start of chemotherapy, serum kappa FLC concentrations had fallen to less than 500 mg/litre and were subsequently maintained below this level. Despite this, the patient remained dialysis-dependent. Another renal biopsy was performed. This showed no evidence of myeloma casts and partial resolution of the inflammatory infiltrate, while the degree of diffuse moderate interstitial fibrosis remained unchanged (Figure 1B).

Both the first and second biopsies were examined by light microscopy in detail along their entire lengths for the presence of casts. Both contained adequate cortical tissue. Tubules in three randomly selected high-power fields from each biopsy were counted. The average number of tubules were 110 for the first biopsy and 121 for the second biopsy per high power field.

The patient had 22 haemodialysis treatments using the high cut-off dialyser. As FLC levels remained below 500 mg/litre, she was switched to a standard high flux schedule. A further 5 days later, 8 weeks from the commencement of chemotherapy, in accordance with the patient's own wishes, haemodialysis was discontinued. Creatinine level at this point was 650 μmol/litre.

At 1 year after discontinuation of dialysis, the patient remains symptomatically well and the myeloma is in remission. Her current eGFR is 8 ml/min/1.73 m^2 ^based on a serum creatinine of 540 μmol/litre, with a maintained urine output. She is clinically well with no symptoms of uraemia and has elected to remain independent of dialysis until she develops clinical symptoms indicative of uraemia.

## Discussion

There are several explanations for resolution of the cast nephropathy. First, resorption via receptor-mediated processes by tubular epithelium and/or mononuclear cells. This is unlikely as the casts represent solid precipitates and distal tubular cells and monocytes have no known receptors for FLC-THP aggregates. Second, tubular epithelial and mononuclear cells might phagocytose cast fragments. This would imply that casts become increasingly friable when FLC concentrations fall below the threshold for further cast formation. But this is not observed histologically. Third, and most likely, tubular cast formation is a dynamic process in patients where there is urine flow. Driven by a high serum light chain load, casts continually precipitate. However, in the presence of sustained glomerular ultrafiltrate, they pass through and out of the intranephronal compartment. Indeed, light chain casts are detected in the urine of patients with cast nephropathy [10]. Therefore, when serum FLC concentrations are reduced, new cast formation ceases and existing casts are flushed out of the tubules. The two biopsies we report may reflect this dynamic process.

At the first biopsy, the histological findings of cast nephropathy, inflammatory cell infiltrate and interstitial fibrosis were concurrent with serum concentrations of serum kappa FLC well above those required to produce cast nephropathy [5]. Serum FLC concentrations dropped by 40% after initial intravenous fluid therapy, indicating that volume contraction secondary to dehydration contributed to the very high presenting FLC concentrations. Rehydration had both a dilutional effect as well as aiding renal clearance, consistent with a maintained urine output. Five days after commencement of the first cycle of dexamethasone, FLC levels had fallen significantly, indicating a strong tumour killing effect. Following commencement of FLC removal by dialysis, the concentrations continued to fall. Five weeks after commencement of dexamethasone, serum FLC concentrations were below 500 mg/litre, the likely minimal threshold for the formation of casts [5].

Whilst cast nephropathy contributes to acute renal failure in myeloma associated with high concentrations of serum FLC, other mechanisms are important. Recent studies have shown that monoclonal FLC are capable of inducing cytoskeletal damage, epithelial-to-mesenchymal transition and apoptosis in proximal tubular epithelial cells in culture, as well as effecting the release of inflammatory cytokines and production of hydrogen peroxide [11-13]. The degree of toxicity and tendency to direct cellular toxicity or cast formation has been shown to be dependent on the clonal origin of the light chain species [11,14]. This direct toxicity may promote the diffuse inflammation and fibrosis that is commonly seen in patients with myeloma kidney [12]. The effects of FLC therefore may be to contribute to direct tubular damage sufficient to produce oliguria through tubulo-glomerular feedback loops. There may then be proximal extension of distal casts and irreversible acute renal failure. The severity of acute renal failure and oliguria therefore may be a product of tubular toxicity, tubular obstruction, dehydration and compounding factors such as the use of diuretics, non-steroidal anti-inflammatory agents and renin-angiotensin system blockade. This may explain why oliguria was not a feature in our patient.

The repeat renal biopsy showed complete resolution of the casts and the interstitial fibrosis remained unchanged from the first biopsy indicating that there was no progression in situ from the time of the initial biopsy. This would be consistent with the observed rapid reduction in FLC concentrations.

In contrast to the case described previously in the literature, our patient did not recover renal function despite rapid, sustained reductions in FLC concentrations and the subsequent disappearance of intratubular casts within 6 weeks of treatment. We speculate there are two reasons for this. First, significant interstitial fibrosis was present in the first biopsy, which developed as a result of exposure to high levels of FLC before presentation. Second, continued exposure to FLC even at concentrations too low to cause cast formation contributed to ongoing inflammation. This would be consistent with incomplete resolution of the inflammatory infiltrate.

The patient was withdrawn from dialysis through personal choice as she was symptomatically well with no uraemic symptoms, despite having an eGFR of 8 ml/min/1.73 m^2^. This is not entirely inconsistent with current practice in the UK, where the average eGFR at initiation of dialysis for individuals of similar age is around 8.4 ml/min/1.73 m^2 ^[15].

Although chemotherapy combined with high molecular weight cut-off haemodialysis effectively reduces the FLC burden on the kidneys, whether renal recovery is achieved or not may depend on the toxicity of the patient's particular light chain clone as well as prompt diagnosis and institution of treatment to reduce FLC concentrations.

## Conclusion

In summary, we believe that this report provides histological evidence that as serum FLC concentrations fall, intratubular casts can resolve within weeks. Early diagnosis and treatment may facilitate reversal of acute renal failure. This observation has important implications for the development of combined haematological and renal treatment strategies aimed at improving renal recovery rates in this setting.

## Abbreviations

eGFR: estimated glomerular filtration rate; FLC: free light chain; H&E: haematoxylin and eosin; MM: multiple myeloma; THP: Tamm-Horsfall protein

## Consent

Written informed consent was obtained from the patient for publication of this case report and any accompanying images. A copy of the written consent is available for review by the Editor-in-Chief of this journal.

## Competing interests

Arthur Bradwell is a major stock shareholder in The Binding Site, Birmingham, UK. FREELITE is produced by The Binding Site, Birmingham, UK. All other authors declare that they have no competing interests.

## Authors' contributions

KB was chiefly responsible for the gathering, analysis and interpretation of data, creation of the figures for this report and was responsible for writing the manuscript. DK and MS performed histological examination of the renal biopsies and were major contributors to the content of the manuscript. CH, NA, MC and HO had significant roles in data gathering, analysis, interpretation and performing critical revisions to the manuscript. AB and PC had a significant role in data gathering, analysis, interpretation and provided significant revisions to the manuscript. All authors read and approved the final manuscript.
